# Preconception-Health-Related Attitudes of Bulgarian Women of Reproductive Age

**DOI:** 10.3390/healthcare11070989

**Published:** 2023-03-30

**Authors:** Eleonora Hristova-Atanasova, Georgi Iskrov, Ralitsa Raycheva, Viktoriya Mandova, Rumen Stefanov

**Affiliations:** Department of Social Medicine and Public Health, Faculty of Public Health, Medical University of Plovdiv, 4002 Plovdiv, Bulgaria

**Keywords:** preconception health and care, reproductive age, women’s health, preconception behavior, weight management, general practitioners (GPs), pre-pregnancy, folic acid, focus group

## Abstract

Preconception care (PC) is relatively new area of practice. While the volume and quality of PC activities depend on local settings, the awareness of women is critical for the successful promotion of PC services. The aim of this study was to examine the preconception-health-related attitudes and experiences of Bulgarian women of reproductive age. A qualitative study conducted among 20 women aged 18 to 49 years was performed between May and July 2022. Two focus groups were used with mixed samples of nulligravida, pregnant, and postpartum women. The participants thought that the Internet and their obstetrician-gynecologist were the only places where they could learn about getting pregnant. Only two of them discussed their PC plans with their physicians. Women pointed out that general practitioners (GPs) need to be more proactive in promoting PC. All respondents outlined the need for a web-based educational platform that could serve as a primary source of health information for future families. The role and functions of GPs in the continuum of PC should be reconsidered. We recommend targeted educational measures for all stakeholders, including women and GPs. In this regard, an easily accessible, knowledge-based web platform could enhance Bulgarian women’s awareness and perceptions of PC.

## 1. Introduction

In Europe, the health of newborns and mothers is an important determinant of the population’s well-being. Even though stillbirth and infant mortality rates have reduced significantly in recent years, they remain a critical public health issue. However, preterm birth, intrauterine growth restriction, and perinatal mortality have also increased with the greater number of women giving birth at a later age [1]. Age is also linked to a higher prevalence of pregnancy problems, including different congenital malformations, hypertension, diabetes, and a higher chance of multiple births. Pregnant women more frequently deliver by caesarean section and are at increased risk of maternal death and morbidity [2,3].

The total number of preconception care (PC) services that are available in each country depends on the local epidemiology, the treatments that are already provided, and the available resources [3,4]. Counselling/health promotion, screening/risk assessment, and treatment/management are the three primary categories into which suggested PC methods are often divided [5,6].

PC is a subject that may be learned through education or experience. According to studies, women who receive prenatal care are more informed and frequently manifest risk-reducing behaviors [6,7]. Women who receive prenatal care also use folic acid more often throughout the advised time frame. A previous study found that women who receive PC interventions are more knowledgeable about prevention strategies, and even brief counselling can increase their understanding of both general and specific preconception health concerns. Murphy et al. indicated that PC is linked to better pregnancy management and a lower chance of negative pregnancy results [7]. Dorney et al. reported that GPs could significantly increase both men’s and women’s access to preconception health counselling and evaluation services [8].

Public health professionals from all over the world are becoming increasingly interested in PC, which is a growing area of research. The PC package should have more than 80 procedures [5]. It should involve a variety of topics, such as family planning, using modern contraception, educating people on safe drug use, reducing drinking and smoking, consuming enough folate and iodine, and managing weight. The goal of PC is to eliminate risk factors that could harm the health of pregnant women and couples. It acts as a gateway in the continuum of health services and treats health issues and prepregnancy concerns that could have detrimental effects on the health of the mother and the newborn [9,10]. Furthermore, congenital malformations can be kept to a minimum [11]. PC also helps families and communities to grow socially and economically [4,5]. Before getting pregnant, less than one-third of women of childbearing age go to a medical professional to talk about their health and how their pregnancy and delivery will go [1,5,12]. Even if a woman of fertility age is not planning a pregnancy, she is still a candidate for PC [4,13]. Many governments have created guidelines and recommendations for PC [14,15,16], and although the WHO announced a PC global agreement, the results of these efforts are still fragmented and inconsistent in most European countries [2].

While there are a few publications that discuss the role of GPs and gynaecologists in family planning, there are no official PC standards in Bulgaria. Furthermore, there is no official health database defined by medical specialists, nor is there a package of services that must be provided during a preconception consultation. The aim of this study was to examine the preconception-health-related attitudes and experiences of Bulgarian women of reproductive age.

## 2. Materials and Methods

### 2.1. Study Setting

Academic staff from the Department of Social Medicine and Public Health at the Medical University of Plovdiv hosted two in-person focus groups. Research on family planning and preconception care has a long history in this department. Moreover, the second-largest metropolis in Bulgaria, Plovdiv, is home to numerous tertiary healthcare facilities and universities.

### 2.2. Research Design

The qualitative study design used a focus group technique to examine respondents’ knowledge, experiences, and opinions [17,18]. As a result, a comprehensive and diverse understanding of women’s attitudes towards preconception care was gained.

### 2.3. Research Participants

Two focus groups of ten participants each were selected based on age, family income, education, employment, and marital status. Both samples included nulligravida, pregnant, and postpartum women. The respondents were selected for the study because they could provide the necessary information about their PC care provision.

Inclusion criteria were as follows: (1) women aged 18 to 49 years; (2) women planning future pregnancies within the next two years; and (3a) women of nulligravida status who were in a relationship or were (3b) pregnant or (3c) up to four years postpartum. Working as a healthcare professional (1) or having a history of unfavorable pregnancy (2) were the exclusion criteria. Each of these two elements reflects extremely specific personal contexts related to education and/or health that had the potential to significantly change the dynamics of the focus group discussion and outcomes.

### 2.4. Sampling and Recruitment

Convenience sampling was applied due to ongoing COVID-19 pandemic restrictions at the time of the study. A total of 37 women volunteered to take part in the survey, 17 of whom dropped out. The latter stated that they saw no advantages or reasons to participate in the focus group discussions. A final sample size of 20 was used with the following distribution: nulligravida (*n* = 9), pregnant (*n* = 1), and postpartum (*n* = 10).

Potential participants were identified and approached through medical consultations and pregnancy and postnatal schools. The moderator booked an individual appointment with each woman to explain the study’s aims and objectives and to provide the survey questions in advance. Based on the availability of the participants, two focus groups were scheduled in May and July 2022.

### 2.5. Ethical Considerations

Approval by an ethics committee was not required for this research. The focus group discussions were sociological in nature and did not include any clinical research or results. No personal information was saved, and only anonymized data were analyzed. Participants provided their written informed consent. This document conforms to the EU General Data Protection Regulation (2016/679) on the protection of natural persons with regard to the processing of personal data and the free movement of such data.

### 2.6. Data Collection

The set of questions was pilot-tested with a group of four women in April 2022. A semistructured discussion included open-ended qualitative items on the following subjects: women’s preconception health knowledge, attitudes, and behaviors; medical professionals’ roles in preconception care; and preconception information sources (Table 1). The same list of questions was used in both focus groups with varying follow-up questions based on the participants’ feedback and reactions. Sociodemographic characteristics were collected at the beginning of each discussion.

### 2.7. Focus Group Discussion Process

The moderator held a separate meeting with every participant prior to the focus group to establish constructive cooperation. At the beginning of the discussion, attendees were reminded about the study’s aim and nature. Each focus group lasted between 90 and 120 min. The discussions were moderated by the first author (a female, MD, PhD), who has expertise with PC, and the second co-author (a male, MPH, PhD), who has experience with qualitative research. Interaction among the participating women was actively encouraged.

The data were originally collected in Bulgarian. Responses were audio-recorded with permission from participants and then transcribed verbatim, while quotes were edited for clarity. Field notes were additionally taken. The collected data were stored in a password-protected file using pseudonyms to preserve anonymity.

### 2.8. Data Analysis

The collected data were analyzed descriptively. By reading the transcripts while listening to the audio recording, the exactness of the transcripts was verified. To ensure the accuracy and trustworthiness of the findings, transcripts were submitted to participants to confirm that they matched the original response and for final approval. The transcripts were then exported for coding, organization, and analysis. Following the six stages described by Braun and Clarke [19,20], the theme analysis method was employed to analyze the data. The six-step process consists of (a) reading over the material many times to facilitate immersion, (b) generating initial codes from the data, (c) sorting various codes to generate themes, (d) evaluating and refining themes, (e) defining and labeling themes, and (f) creating a clear, cohesive, and logical report with data extracts. The co-authors coded and validated the data. The Consolidated Criteria for Reporting Qualitative Research (COREQ) was followed to report the findings (Supplementary Table S1) [21]. No software package was used for the qualitative data analysis.

### 2.9. Trustworthiness

In parallel with the conventional quantitative assessment criteria, the validity and reliability of the present study were guaranteed by applying Lincoln and Guba’s [22] refined trustworthiness concept for credibility, transferability, dependability, and confirmability.

## 3. Results

Four major themes, (1) profiles of women; (2) PC knowledge, attitudes, and behaviors; (3) medical professionals’ roles in PC; and (4) women’s information sources, and nine subthemes were derived from the data about the provision of, and perceptions regarding, PC. Table 2 provides an overview of the main themes and subthemes.

### 3.1. Theme 1: Profiles of Women 

The two subthemes of this chapter consist of sociodemographic and risk behavior profiles. By utilizing these profiles, we may assess a woman’s PC knowledge and attitude in relation to her personal life and preconception practices.

#### 3.1.1. Subtheme 1.1: Sociodemographic Profile

The mean age was 28.3 years (range: 22–38), and approximately 80% of the women had a master’s degree; more than 50% were employees. The mean age at first childbirth was 27.1 years (range: 20–37), with nine women having one child and two having multiple children. Only one respondent was divorced; the rest were married at the time of giving birth. Table 3 shows the distribution of participants’ characteristics by age, family income, education, employment, and marital status.

#### 3.1.2. Subtheme 1.2: Risk Behavior Profile

We assessed the women’s PC behaviors and attitudes based on their lifestyles and activities. We asked the participants to specifically refer to their personal perceptions and knowledge at the time at which a family planning decision was made. This time point for nulligravida was at the present moment of the study, while pregnant and postpartum women referred to prepregnancy experiences.

None of the women listed preventative measures as a priority for their maternal health. The mean BMI was 24.0 kg/m^2^ (range: 17.7–32.6), but none of the women were consuming a well-balanced diet, they did not consume the daily recommended intakes of fruits and vegetables, and were drinking more than the recommended daily dose of caffeine. The surveyed women were involved in limited physical activity, and 13 of them smoked or were smoking before and after pregnancy. Only one of the postpartum women reported having a chronic illness prior to pregnancy as well as a family history of genetic malformations (no other details were requested to protect the participant’s privacy). The only medications used were vitamins and folic acid, but not all women, regardless of their status, had taken both or either of the supplements. Three of the respondents worked in a hazardous environment, and 20% of them had experienced previous miscarriages.

### 3.2. Theme 2: PC Knowledge, Attitudes, and Behaviors

Three subthemes emerged regarding participants’ perceptions of PC: (1) PC as a strategy for prevention and family planning; (2) appropriate folate intake; and (3) addressing health issues prior to pregnancy.

#### 3.2.1. Subtheme 2.1: PC and Family Planning

The findings identified the presence of several barriers and a lack of knowledge regarding preconception care. Women’s interest in this subject was to learn more in case they became pregnant in the future. None of the respondents were familiar with the phrase “preconception care”, and none of them were aware of exactly what it entails. The vast majority of attendees were intending to become pregnant. This timeframe was from 2 months for a future planned pregnancy up to 4 years for two women who had used in vitro fertilization (IVF).

*Participant 13: “I have no idea what is meant by the phrase “preconception care” because I have never come across it before.”*

*Participant 20: “My wedding planning coincided with our initial discussions about when we should have a baby. After the honeymoon, I discovered that I was pregnant.”*

#### 3.2.2. Subtheme 2.2: Folate Intake

The respondents considered the lack of access to information or inaccurate information provided by obstetrician-gynaecologists (OB-GYNs) as the main reason for why women do not take folic acid or start taking it too late. None of them were aware of the purpose of consuming folic acid. The information source (OB-GYN, friends, or the Internet) was found to essentially determine the dose and administration time.

*Participant 5: “I haven’t received a folic acid prescription from a doctor. After my child was born, I found out from other mothers that they had taken folic acid while they were pregnant.”*

*Participant 14: “We have been trying to conceive for the past four months. However, I was never advised to take folic acid by anyone.”*

#### 3.2.3. Subtheme 2.3: Health Issues Prior to Pregnancy

All respondents agreed that the reduction and elimination of risk factors are important to prevent complications during conception, pregnancy, and birth. Nevertheless, when asked to identify specific risk factors, the attendees only mentioned obesity, alcohol consumption, and smoking. No one mentioned hazardous environments, the impact of genetic disorders in families, or infectious diseases and immunizations as part of preconception care.

*Participant 8: “I have many overweight friends who had pregnancy complications. Maintaining a healthy weight balance for both partners is critical for a healthy generation.”*

*Participant 1: “I am an anti-vaxxer and would not immunise myself prior to or during pregnancy, nor will I vaccinate my child in the future.”*

### 3.3. Theme 3: The Medical Professional’s Role in PC

This theme contains two subthemes depending on which medical professional must deliver PC.

#### 3.3.1. Subtheme 3.1: Obstetrician-Gynaecologists (OB-GYNs)

OB-GYNs were found to be the primary source of information for women of childbearing age. Only two of the participants had discussed future pregnancy plans with their physicians. According to them, because of anxiety and shyness, the majority of women of reproductive age seek medical care too late.

*Participant 4: “I didn’t visit my GP to discuss a future pregnancy. I went to the obstetrician when I found out I was pregnant. The doctor was a reliable source of information for me.”*

*Participant 7: “My OB-GYN has always made me feel comfortable discussing my sexual health and family planning options, including asking me when I’m going to start a family.”*

#### 3.3.2. Subtheme 3.2: General Practitioners (GPs)

A large number of women stated that general practitioners (GPs) should be more involved in providing preconception care. Women also pointed out that GPs need to be more proactive in promoting preconception care through individual or group family planning counselling and/or the distribution of educational materials to every woman of reproductive age. A high percentage of nulligravida women reported severe difficulty in obtaining appointments with their primary care physicians. They did not feel the need to consult a medical practitioner about family planning and future pregnancies. They obtain this information primarily from the Internet. They only go to their general practitioner when they become sick.

*Participant 3: “I would ask my GP, but that’s not her area of expertise, and she is not so informed of the benefits of folate intake.”*

*Participant 9: “I go to my GP when I’m sick, but not to discuss family planning.”*

*Participant 7: “If I needed information about a future pregnancy, I would probably just google it or just ask a friend of mine instead of going to my GP.”*

### 3.4. Theme 4: Women’s Information Sources

This section’s subthemes concern the Internet and other information sources identified by female respondents.

#### 3.4.1. Subtheme 4.1: Internet Sources

The women who were interviewed thought that the Internet and OB-GYNs were the only places for learning about getting pregnant. All respondents outlined the need for a web-based educational platform that could serve as a primary source of health information for future families.

*Participant 5: “I think that an up-to-date website should be made to help women before they get pregnant. Many of my friends are embarrassed to talk to a medical specialist.”*

*Participant 19: “Until now, my only sources of information on conception, contraception, and parenthood were the internet and my OB-GYN.”*

*Participant 15: “It would be good to have a public website where ladies may share their prenatal struggles.”*

#### 3.4.2. Subtheme 4.2: Other Sources

Women acknowledged a lack of general education about, and research on, the consumption of a healthy diet and folate intake, stating that more information should be disseminated about this topic in schools and by medical experts. They also suggested that medical professionals could lead open discussions and online seminars to help them learn more about all aspects of preconception care.

*Participant 11: “Before a possible second pregnancy, I would participate in preconception care webinars so that I could ask questions of experts with difficult appointment schedules.”*

*Participant 18: “Healthcare professionals should be involved in educating students about lifestyle factors that affect infertility or high-risk pregnancies. Additionally, future generations should be taught about effective methods of birth control.”*

## 4. Discussion

The study demonstrates the various preconception care challenges faced by Bulgarian women as well as their limited preconception health knowledge. This qualitative research offers a variety of women’s perspectives about their own reproductive behaviors. Despite the small number of participants recruited for this study, the findings clearly illustrate the need for preconception care strategies and medical standards to be put into practice. These results are supported by additional research on the subject [23,24,25,26,27,28,29].

Information about the attitudes and behaviors of Bulgarian women of reproductive age on topics and methods related to preconception care is scarce to nonexistent. Preconception care is now a routine practice in many European countries due to the increased prevalence of obesity, type 2 diabetes, and delayed childbearing, which raise the risk of pregnancy complications and unfavorable outcomes [30,31].

Based on our findings, a high number of women of reproductive age are continuing to expose themselves to risk factors like caffeine, alcohol, and cigarettes. According to a population survey, 98% of couples trying to conceive have at least one risk factor that could be addressed [32,33]. There is growing evidence from observational and epidemiological studies that hazardous environmental exposures are linked to reproduction issues. As a result, the early identification of high-risk groups of women is critical for preconception care. It is vital to keep these women aware of the potential risk of adverse pregnancy outcomes associated with these exposures [34,35].

During the preconception period, the majority of our respondents stated that they did not follow a balanced dietary regimen, and they reported insufficient physical activity levels. However, the participants themselves identified these two issues as health concerns that need to be resolved prior to conception. These statements are consistent with the growing interest in providing a global intervention to detect and eliminate various risk factors, including obesity, chronic diseases, and not consuming the vitamin folic acid before pregnancy [24,36,37,38]. This is in agreement with findings from other countries, where more than half of respondents stated that they exercise less than twice per week and only about 20% of the studied population were consuming a well-balanced diet [39,40,41,42]. Furthermore, a cross-sectional survey of attitudes about lifestyle, fertility, and pregnancy in women and men from 104 countries revealed that the consumption of a variety of fruits and vegetables as well as abstaining from alcohol and smoking were viewed as the most essential lifestyle habits for the promotion of healthy childbirth. Given the high prevalence of overweight and obesity and the detrimental consequences of having excess weight on outcomes in the mother and newborn, prioritizing a healthy lifestyle before conception should be encouraged [43,44].

Most of the women in our study were aware of the value of consuming folic acid during pregnancy, but very few of them took supplements for the entire recommended periconceptional period. Surveys from different countries have yielded similar findings, revealing that folic acid intake is less essential for healthy childbearing [39,43,44,45]. On the other hand, the Australian Longitudinal Research on Women’s Health found that many women take at least one dietary supplement before becoming pregnant [46]. In Bulgaria, a national study of the nutrition of newborns and young children up to the age of 5 years found that only 4% of the mothers involved took folic acid before becoming pregnant. Our government’s recommendation that women of childbearing age start taking folic acid supplements before getting pregnant do not appear to be highly effective for preventing neural tube defects (NTDs) [47,48,49]. Individual or group consultations with medical professionals as well as general education on the importance of folic acid could help to increase uptake.

The majority of the participants intended to become pregnant in the future but demonstrated a limited understanding of the meaning and goals of preconception care. Conception, according to them, begins with a positive pregnancy test result. At that point, their attitudes begin to shift, and they become extremely concerned about the health of their fetus. The period between conception and pregnancy diagnosis, on the other hand, is one of uncertainty. Women’s behaviors would shift if they were given the necessary information and education throughout this period [29,50,51].

Poels et al. identified numerous advantages and challenges associated with preconception consultation. Worry and anxiety were highlighted as difficulties as well as the time and effort required to contact a doctor for consultation [35]. Furthermore, GPs play a crucial role in determining the best way to provide preconception care and allocating resources to properly support and provide this service. In our study, two controversial positions were expressed. Some of the women claimed that the lack of GP preconception care initiatives indicates the need for a more proactive role. Meanwhile, some women did not feel the need to consult a medical practitioner about family planning and future pregnancies. According to a review by Shannon et al., primary care is the most common location at which preconception health services are provided. The authors, however, concluded that there is no universally accepted consensus on the best way to administer treatment in primary care. To improve service delivery, it is likely that a combination of methods will be required. This is consistent with the results of a qualitative study that revealed that women who take a micronutrient supplement are more inclined to make investments in their preconception health and care [52,53].

The Internet is considered the primary source of information, particularly for fertility issues, and it emerges from both contexts that women in the preconception period are concerned about how to enhance conception. There is an increasing number of web-based platforms dedicated to preconception health across the world [54], but none are produced in Bulgaria. Accurate and reliable preconception health information may eventually contribute to the prevention of unfavorable pregnancy outcomes. Finally, without sufficient understanding, young people will be unable to make informed decisions about childbearing and sexual and reproductive health.

The highly educated cohort of mostly Bulgarian ethnicity are limitations that may restrict the external validity of the findings. Another argument is that the majority of participants were very willing and motivated to have children in the future. Even though the sample size was small, it was comparable to previous studies on similar topics [29,32] and is optimal for reaching theoretical saturation.

## 5. Conclusions

It is essential to account for the attitudes and opinions of women in order to build an evidence-based, sustainable strategy for reproductive health. In this regard, our study revealed important opportunities for improvement in the provision of PC as well as the promotion of health behaviors among women. We found the combination of a low level of PC knowledge and reliance on untrustworthy information sources on the Internet to be key obstacles to the efficient implementation of PC in Bulgaria. All focus group participants were largely unaware of the role and impact of major PC risk factors on potential pregnancies and child health. The PC activities that are currently offered by Bulgarian GPs were deemed to be unsatisfactory and superficial.

We recommend targeted educational measures for all stakeholders, including women and GPs. In this context, an easily accessible, knowledge-based web platform could enhance Bulgarian women’s awareness and perceptions of PC. Such a health promotion tool could also be used by local health authorities to provide comprehensive recommendations about PC and reproductive health. The role and functions of GPs in the continuum of PC should be reconsidered. It is necessary to allocate more resources to primary healthcare in order to facilitate access to medical consultations and primary prevention services.

## Figures and Tables

**Table 1:** List of questions used in the focus group.

Subject	Questions
PC knowledge	What triggered your interest in this subject? To date, what has been your experience with PC? Was your pregnancy planned or will you be planning it? What do you know about folate and pregnancy? Is dietary folate adequate for meeting women’s needs during pregnancy? How much folate (mg) should women take and when should they take it to prevent neural tube defects? What other health issues need to be addressed prior to pregnancy?
PC attitudes and behaviors	In your opinion, why do women not take folic acid or start taking it too late? What would encourage women to use folic acid before conception? With whom have you spoken regarding your intention to have a baby? Why? What did you expect? What did he or she tell you? What was his or her attitude towards you? Were there any questions you wanted to ask but, for some reason, did not? Why?
Medical professionals’ role in PC	Why do so many women only visit an OB-GYN after becoming pregnant? What is the role of a GP in PC? Did the GP provide you with PC on his own initiative? How should GPs discuss PC issues with women?
Information Sources about PC	Have you ever sought information on the Internet about conception and pregnancy? What have you searched for? Have you looked at any other information about getting pregnant or getting ready to get pregnant? What kind of information have you obtained from these sources? What future pregnancy-related topics do you think should be on a web-based health promotion platform? Do you have any other suggestions, questions, or recommendations?

**Table 2:** Summary of the main themes and subthemes.

Themes	Subthemes
PROFILES OF WOMEN	Sociodemographic profile
	Risk behavior profile
PC KNOWLEDGE, ATTITUDES, AND BEHAVIORS	PC and family planning
	Folate intake
	Health issues prior to pregnancy
MEDICAL PROFESSIONAL’S ROLES IN PC	Obstetrician-gynaecologists (OB-GYNs)
	General practitioners (GPs)
WOMEN’S INFORMATION SOURCES	Internet sources
	Other sources

**Table 3:** Participants’ characteristics.

N	Women	Age	Employment Status	Education Degree	Family Income (BGN)	Marital Status
1	nulligravida	22	unemployed	high school	1000–2000	living together
2	pregnant	34	employee	master’s	1000–2000	married
3	nulligravida	23	employee	master’s	≥2000	married
4	postpartum	30	self-employed	master’s	1000–2000	married
5	postpartum	38	employee	master’s	≥2000	married
6	postpartum	28	employee	high school	≥2000	married
7	nulligravida	22	unemployed	high school	1000–2000	living together
8	nulligravida	28	employee	master’s	1000–2000	living together
9	postpartum	31	self-employed	master’s	≥2000	living together
10	postpartum	26	employee	master’s	1000–2000	divorced
11	postpartum	36	employee	master’s	≥2000	married
12	nulligravida	26	employee	master’s	1000–2000	living together
13	nulligravida	23	employee	master’s	≥2000	married
14	nulligravida	26	employee	master’s	1000–2000	living together
15	nulligravida	28	employee	master’s	1000–2000	living together
16	nulligravida	36	employee	master’s	≥2000	married
17	postpartum	27	unemployed	master’s	1000–2000	married
18	postpartum	23	unemployed	high school	≥2000	living together
19	postpartum	29	employee	master’s	≥2000	married
20	postpartum	30	unemployed	high school	1000–2000	living together

## Data Availability

Data will be provided on request.

## Supplementary Materials

The following supporting information can be downloaded at: https://www.mdpi.com/article/10.3390/healthcare11070989/s1, Table S1. COREQ (COnsolidated criteria for REporting Qualitative research) Checklist.
